# Synthesis and Electronic Structure of Mid-Infrared Absorbing Cu_3_SbSe_4_ and Cu_*x*_SbSe_4_ Nanocrystals

**DOI:** 10.1021/acs.chemmater.3c00911

**Published:** 2023-08-09

**Authors:** Annina Moser, Olesya Yarema, Gregorio Garcia, Mathieu Luisier, Filippo Longo, Emanuel Billeter, Andreas Borgschulte, Maksym Yarema, Vanessa Wood

**Affiliations:** † Institute for Electronics, Department of Information Technology and Electrical Engineering, ETH Zurich, Gloriastrasse 35, CH-8092 Zurich, Switzerland; ‡ Departamento de Tecnología Fotónica y Bioingeniería & Instituto de Energía Solar, ETSI Telecomunicación, Universidad Politécnica de Madrid, Ciudad Universitaria, ES-20840 Madrid, Spain; § Institute for Integrated Systems, Department of Information Technology and Electrical Engineering, ETH Zurich, Gloriastrasse 35, CH-8092 Zurich, Switzerland; ∥ Laboratory for Advanced Analytical Technologies, Empa, Überlandstrasse 129, CH-8600 Dübendorf, Switzerland; ⊥ Department of Physics, Danmarks Tekniske Universitet, Fysikvej, Building 312, 2800 Kgs. Lyngby, Denmark; # Department of Chemistry, University of Zurich, Winterthurerstrasse 190, CH-8057 Zürich, Switzerland

## Abstract

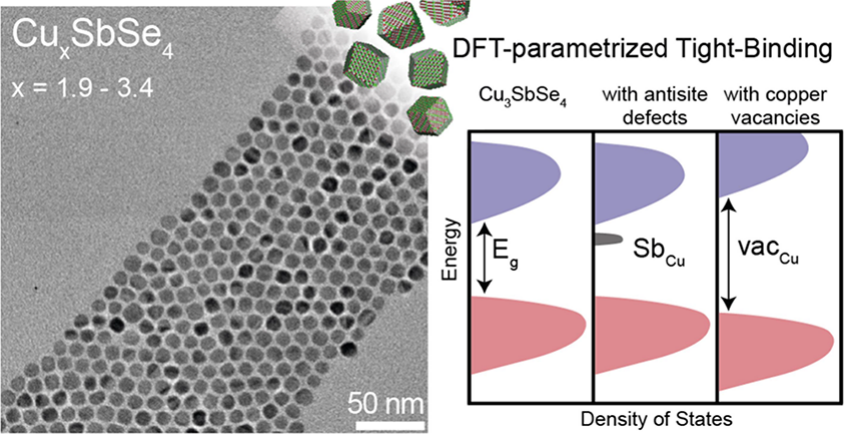

Aliovalent I–V–VI semiconductor nanocrystals are promising candidates for thermoelectric and optoelectronic applications. Famatinite Cu_3_SbSe_4_ stands out due to its high absorption coefficient and narrow band gap in the mid-infrared spectral range. This paper combines experiment and theory to investigate the synthesis and electronic structure of colloidal Cu_*x*_SbSe_4_ nanocrystals. We achieve predictive composition control of size-uniform Cu_*x*_SbSe_4_ (*x* = 1.9–3.4) nanocrystals. Density functional theory (DFT)-parametrized tight-binding simulations on nanocrystals show that the more the Cu-vacancies, the wider the band gap of Cu_*x*_SbSe_4_ nanocrystals, a trend which we also confirm experimentally via FTIR spectroscopy. We show that Sb_Cu_ antisite defects can create mid-gap states, which may give rise to sub-bandgap absorption. This work provides a detailed study of Cu_*x*_SbSe_4_ nanocrystals and highlights the potential opportunities as well as challenges for their application in infrared devices.

## Introduction

Ternary I–V–VI materials (group I—Ag, Cu; group V—Sb, Bi; group IV—S, Se, Te) exhibit unique properties such as vibrational anharmonicity and superior absorption properties due to the lone electron pair of pnictogen.^1,2^ Famatinite Cu_3_SbSe_4_ is a promising candidate for thermoelectric and mid-infrared active photoelectronic devices. Several studies investigate its applicability for thermoelectrics due to particularly low thermal conductivity and high electrical conductivity.^3^ Furthermore, Cu_3_SbSe_4_ is a direct band gap semiconductor with the absorption onset around 0.2 eV,^3^ which suggests that it could be used as a mid-infrared absorber material.^4^

Colloidal nanocrystals of I–V–VI compositions have seen rapid development of synthesis protocols, characterization, and device integration.^5,6^ For example, AgBiS_2_ nanocrystals have been used in solar cells.^7^ Earth-abundant Cu–Sb–S and Cu–Sb–Se phases show absorption coefficients above 10^5^ cm^–1^ at a wavelength of 400 nm and band gaps in the infrared.^2,8,9^ Cu_3_SbS_4_ has a band gap *E*_g_ = 1.0 eV and good photoelectric response.^10^ Recently, developed recipes for colloidal nanocrystals of various Cu–Sb–S and Cu–Sb–Se phases further expand the scope of characterization and application.^11,12^

Among these, colloidal Cu_3_SbSe_4_ nanocrystals remain little studied.^13^ In our previous paper, we investigated the synthesis and optical properties of multiple I–V–VI selenide nanocrystals, finding that Cu_3_SbSe_4_ nanocrystals are the most absorptive of the I–V–VI colloids.^12^ A study of nanocrystal thin films reveals intrinsic *p*-type doping, a significant carrier density increase upon ambient exposure, and a strong dependence of conductivity on surface ligands.^14^

Compared to binary compositions, multi-cationic nanocrystals allow for greater tunability of properties such as photoluminescence or electronic transport properties;^15,16^ however, their structure is more complex. Cation disorder and vacancy formation further increase structural complexity.^17−19^ Computational modeling elucidates the effects of atomic ordering on experimentally observable properties,^20^ and how electronic and optical properties can be altered by the introduction of vacancies,^21^ targeted doping,^22,23^ and secondary phases.^24,25^

While the electronic structure of Cu_*x*_SbSe_4_ nanocrystals with *x* ≤ 3 has not yet been explored, first-principles calculations performed on various types of point defects in bulk famatinite Cu_3_SbSe_4_ revealed a low formation energy of Cu-vacancies,^26^ suggesting that Cu vacancies will be prevalent compared to other defect types. This small concentration of Cu vacancy defects for slightly non-stoichiometric bulk Cu_*x*_SbSe_4_ (2.97 ≤ *x* < 3)^27^ leads to band splitting, valence band states above the Fermi energy, and intrinsic *p*-type doping.^14^

Here, we present a systematic study of the synthesis and defect chemistry of famatinite-type Cu_*x*_SbSe_4_ nanocrystals and its impact on the electronic structure and optical properties. By exploring the reaction time, temperature, and precursor concentration, we gain insight into the reaction mechanism and achieve size uniformity and composition control for Cu_*x*_SbSe_4_ nanocrystals, with *x* = 1.9–3.4. With density function theory-parametrized tight-binding simulations, we calculate the electronic structure for stoichiometric Cu_3_SbSe_4_ nanocrystals, non-stoichiometric Cu_*x*_SbSe_4_ nanocrystals with Cu-vacancies, and nanocrystals with Cu_Sb_ and Sb_Cu_ antisite defects. Our findings explain the experimentally observed trends in infrared absorption for Cu_*x*_SbSe_4_ nanocrystals.

## Experimental Methods

### Materials

Antimony(III) chloride (99.999%, STREM), copper(I) chloride (99.99%, Sigma-Aldrich), 1-dodecanethiol (DDT, 98%, Sigma-Aldrich), oleylamine (OLA, techn. 80–90%, Acros), selenium (shots, 99.99%, STREM), chloroform (anhydrous, >99%, Sigma-Aldrich), ethanol (anhydrous, 99.9%, Acros), tetrachloroethylene (anhydrous, 99%, Sigma-Aldrich), tetrabutylammonium iodide (TBAI, 99%, Sigma-Aldrich), and methanol (anhydrous, 99.8%, Sigma-Aldrich) were used.

### General Remarks on Synthesis

All syntheses are carried out in an air-free environment using standard Schlenk line technique. Oleylamine is purified from water residues by heating to 100 °C under vacuum for at least 1 h. The solvents are then transferred into a N_2_-filled glovebox. All other chemicals are used as purchased. Injection mixtures and stock solutions are prepared in the glovebox. A stock solution of 0.1 M CuCl is prepared by dissolving respective amounts in oleylamine at 70 °C. A stock solution of 0.5 M selenium in OLA/DDT is prepared by dissolving elemental Se at 100 °C in the respective solvents with a volume ratio of 1:1.

### Synthesis

In accordance with the previously published synthesis,^12^ Cu_*x*_SbSe_4_ nanocrystals are prepared by dissolving the desired amount of SbCl_3_ in dried oleylamine at 70 °C in the glovebox. Together with the respective amount of 0.1 M CuCl stock solution, the SbCl_3_ precursor is loaded to the three-neck flask connected to the vacuum manifold and heated under vacuum for another 30 min. After backfilling the flask with nitrogen and reaching the chosen set temperature between 70 and 200 °C, 0.5 M Se stock solution is swiftly added. Upon reaching the reaction time, the crude solution is rapidly cooled with a water bath. The nanocrystals are purified using a standard solvent/non-solvent procedure and stored in chloroform under inert atmosphere.

### Basic Characterization

TEM images are acquired on a Hitachi HT7700 and on a JEOL JEM-1400 Plus, both operating at 100 keV. High-resolution TEM and STEM/EDX images are taken on a FEI Talos operating at 200 keV. For better STEM/EDX imaging, TEM samples are submerged in ethanol after drop-casting the nanocrystal solutions on TEM grids. Size distributions are evaluated by measuring >100 particles per sample with ImageJ software. Absorption spectra are measured with an Agilent Cary 5000 UV–Vis–NIR spectrophotometer by measuring diluted nanocrystals in tetrachloroethylene. For absorption coefficient determination, a thin film of nanocrystals with known thickness is spin-coated on a glass substrate and transmission and reflection is determined. Fourier transform infrared spectroscopy (FTIR) measurements are performed on a Bruker Vertex 70 spectrometer at room temperature by drop casting nanocrystal solutions on ZnSe windows. Absorption spectra from different measurements are joined together by matching overlapping energy ranges. Energy-dispersive X-ray spectroscopy (EDX) data are measured with FEI Quanta 200 FEG SEM microscopes, operating at 30 keV. X-ray diffraction (XRD) measurements are carried out on a Rigaku SmartLab 9 kW System with a rotating Cu anode and a HyPix-3000SL 2D solid-state detector. Rietveld refinement is performed with FullProf Suite software.

### Thin Film Fabrication

For nanocrystal thin films, the colloidal solutions are purified once more with ethanol, centrifugation, and redissolving in chloroform. Filtered solutions are dropped on clean substrates and spin coated to form a single monolayer (approx. 15 nm thickness). Films are soaked for 30 s with TBAI solution in methanol (20 mg/mL) to remove surface ligands and subsequently spin-washed three times with pure methanol. For thicker films, the deposition cycle is repeated on top of a previous layer. The thickness is measured with an Agilent 5500 Atomic force microscope in tapping mode.

### X-ray Photoelectron Spectroscopy (XPS)

For XPS measurements, 2 nm Cr and 30 nm Au are evaporated on undoped Si substrates. A single layer of nanocrystals is spin coated according to the recipe described above. XPS analysis is performed using a PHI Quantes spectrometer (ULVAC-PHI), as equipped with a conventional low-energy Al-Kα source (1486.6 eV) and a high energy Cr-Kα (5414.7 eV) X-ray source. Both sources are high flux focused monochromatic X-ray beams that can be scanned across the sample surface to analyze a selected area on the sample surface. The energy scale of the hemispherical analyzer is calibrated according to ISO 15472 by referencing the Au 4*f*_7/2_ and Cu 2*p*_3/2_ main peaks (as measured in situ for corresponding sputter-cleaned, high-purity metal references) to the recommended binding energy (BE) positions of 83.96 and 932.62 eV, respectively. A dual beam charge neutralization system is applied during each measurement cycle, employing low energy electron and argon ion beams (1 V bias, 20 μA current). XPS survey spectra, covering a BE range from 0 to 1100 eV, are recorded with a step size of 0.4 eV at a constant pass energy of 112 eV using the Al-Kα source (power 24.5 W; beam diameter 100 μm). XPS detail regions (i.e., Cu 2*p*, Se 3*d*, O 1*s*/Sb 3*d*, and Sb *MNN*) are recorded with a step size of 0.05 eV at a constant pass energy of 55 eV using the same as above Al-Kα source. Chemical state analysis of O 1*s*/Sb 3*d* as well as Cu 2*p* and Se 3*d* regions is performed using CasaXPS 2.3.22PR1.0 software,^28^ employing a GL(30) lineshape for peak fitting of O 1*s*, Se 3*d,* and Sb 3*d* and a GL(80) lineshape for Cu 2*p*. The fitting procedure is constrained by a spin–orbit splitting of 9.34 eV for the Sb 3*d* peaks and an area ratio of 0.66 between Sb 3*d*_3/2_ and Sb 3*d*_5/2_, 0.9 eV and 0.66 between Se 3*d*_3/2_ and Se 3*d*_5/2_, and 0.5 between Cu 2*p*_1/2_ and Cu 2*p*_3/2_.^29^

### Theory Calculations

Density functional theory (DFT) calculations for famatinite Cu_3_SbSe_4_ structure were described in detail previously.^3^ Here, a tetragonal unit cell with 16 atoms (Cu_6_Sb_2_Se_8_) is modeled with structural data from the CCL crystallographic database.^30^ To create a tight-binding parameter set from the DFT bands of Cu_3_SbSe_4_, a fitting algorithm relying on least-square minimization is used. The three highest valence bands and two lowest conduction bands are included in the procedure. A *sp*^3^*d*^5^ orbital combination is chosen for the Cu atoms, while the 5*d* orbitals are replaced by an excited s* orbital for Sb and Se. Further discussions of the fitting and resulting parameters are given in Supplementary Information. Nanocrystal structures are created by extending the relaxed unit cell to a bulk structure and then carving out a crystal with well-defined cutting planes along (110). Tight-binding simulations are performed with the atomistic and full-band quantum transport simulator OMEN^31^ using 25 eV *dsp*^3^ hybridization for dangling bonds.^32^ The output includes eigenmodes with corresponding energy values around the band gap as well as their respective wave functions and the optical coupling matrix. The OMEN results are evaluated with Mathematica and MATLAB software.

## Results and Discussion

### Synthesis of Cu_*x*_SbSe_4_ and Cu_3_SbSe_4_ Nanocrystals

**Scheme 1:**
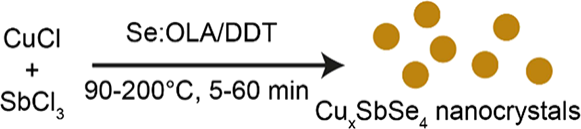
Synthesis Route for Cu_*x*_SbSe_4_ Nanocrystals

To obtain high-quality Cu_*x*_SbSe_4_ nanocrystals, we employ hot-injection synthesis.^12,13^ Elemental Se dissolved in a mixture of oleylamine and dodecanethiol forms a highly reactive polyselenide reagent.^33^ This Se precursor is then injected in a hot oleylamine-based mixture of Cu and Sb chlorides, leading to the burst nucleation and growth of Cu_*x*_SbSe_4_ colloids (Scheme 1).

**Figure 1:**
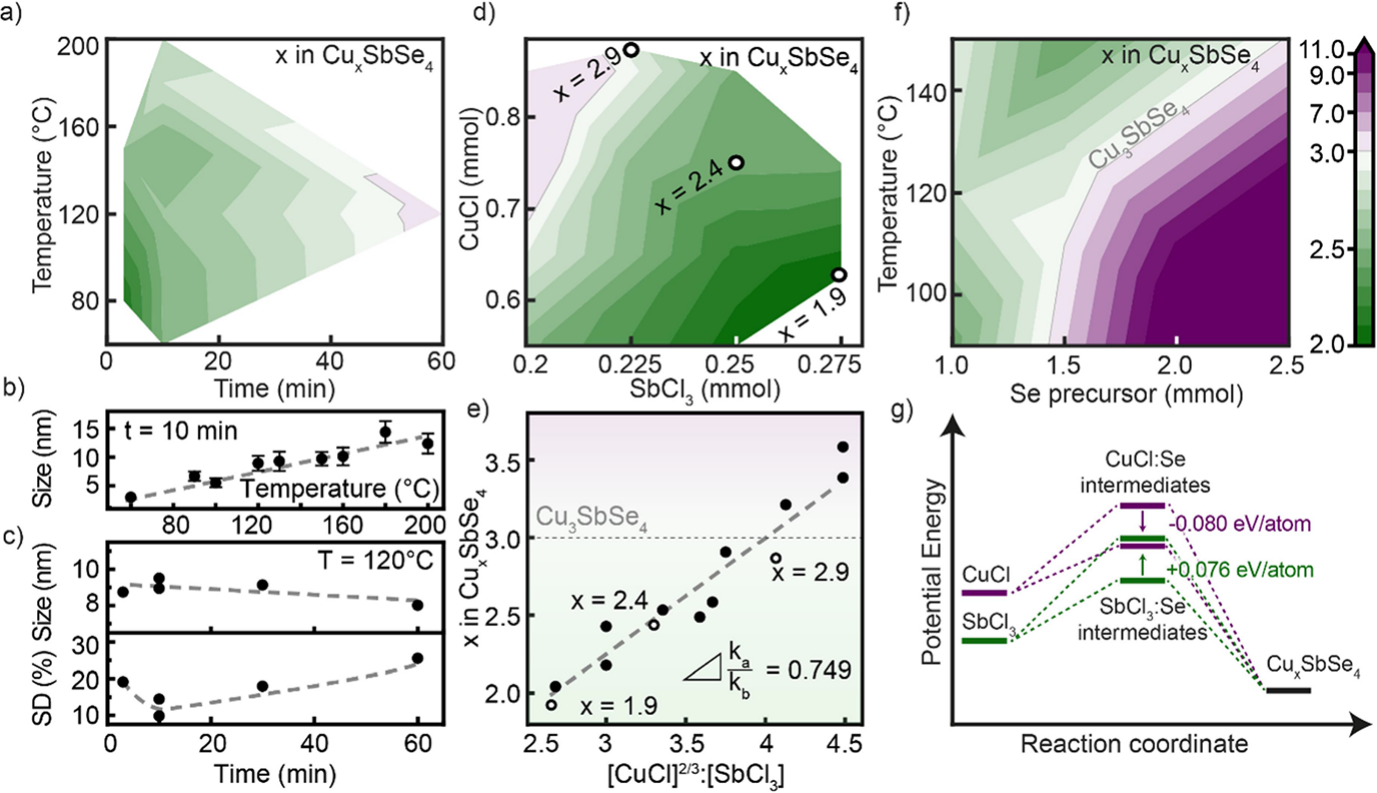
(a) Experimental composition maps for Cu_*x*_SbSe_4_ nanocrystals, synthesized at different reaction temperatures and growth times (0.75 mmol CuCl; 0.25 mmol SbCl_3_; 1 mmol Se). (b) Size of Cu_*x*_SbSe_4_ nanocrystals (shown in panel a) at 10 min prepared at different reaction temperatures. (c) Size and size distribution of Cu_*x*_SbSe_4_ nanocrystals (shown in panel a) at 120 °C as a function of different growth times. (d) Experimental composition maps for Cu_*x*_SbSe_4_ nanocrystals, starting from different amounts of CuCl and SbCl_3_ (10 min reaction at 130 °C). (e) Dashed line indicates composition of Cu_*x*_SbSe_4_ nanocrystals as a function of halide amounts to the power of partial reaction orders (here, ^2^/_3_ for CuCl and 1 for SbCl_3_). Actual compositions are shown with data points; open data points indicate the three Cu_*x*_SbSe_4_ compositions *x* = 1.9, *x* = 2.4, and *x* = 2.9 that are extensively studied. (f) Composition of Cu_*x*_SbSe_4_ nanocrystals as a function of Se precursor amount and reaction temperature (10 min reaction with 0.75 mmol CuCl and 0.25 mmol SbCl_3_). (g) Proposed reaction coordinate diagram for the formation of Cu_*x*_SbSe_4_ nanocrystals. Arrows indicate the change in activation energy coming from an excess of Se precursor.

To determine the optimal growth parameters for uniform composition and low size distribution, we start our investigation of the Cu-Sb-Se colloidal synthesis with a parameter sweep of injection temperature and growth time (Figures 1a and S1). For these experiments, we keep stoichiometric amounts of elemental precursors (i.e., the molar ratio of CuCl:SbCl_3_:Se is 3:1:4), and measure the size, size distribution, and composition of the obtained Cu–Sb–Se nanocrystals. In agreement with previous work on other I–V–VI materials,^12^ higher temperature leads to faster growth kinetics and hence to a larger diameter of nanocrystals (Figure 1b). By choosing the reaction temperature from 60 to 220 °C, the size of Cu–Sb–Se nanocrystals can be controlled in the range of 3–15 nm. Growth time, however, has little influence on the average size of nanocrystals (Figure 1c), suggesting a reaction, where at least one of the precursors is fully consumed during the synthesis. Analysis of the size distribution over time (Figure S1b) provides further details of the formation of nanocrystals. We observe size focusing up to a growth time of approximately 10 min and broader size distributions for longer reaction times (Figure 1c). The size focusing phenomenon has been associated with high supersaturation (*S* ∼ 100) of initial precursors in the reaction mixture.^34^ At such abundance of reactants, all nucleate centers have the same volume growth rate; therefore, smaller nanocrystals increase faster in diameter. The increase in size distribution for reaction times longer than 10 min indicates that 10 min marks the onset of Ostwald ripening growth, characterized by the lack of one or more elemental precursors.^34^

The composition map for the sweep of time and temperature (Figure 1a) reveals that at optimal growth time of 10 min, the nanocrystals are Cu-deficient. With ∼15–20% of Cu atoms missing, the composition of nanocrystals can be presented as Cu_*x*_SbSe_4_, where *x* = 2.4–2.6. This composition map suggests that the Sb precursor is fully consumed after 10 min, while remaining unreacted CuCl continues to decompose, allowing Cu to slowly diffuse into the nanocrystals. Stoichiometric Cu_3_SbSe_4_ nanocrystals are achieved through long reaction times; however, under these conditions, Cu_3_SbSe_4_ nanocrystals have large size distributions.

With the goal of achieving stoichiometric and monodisperse Cu_3_SbSe_4_ nanocrystals, we proceed to systematically adjust the concentration of cation precursors, while retaining the optimal time from previous experiments (i.e., 10 min) and constant temperature of 130 °C (Figure 1d). We achieve stochiometric Cu_3_SbSe_4_ nanocrystals as well as compositions with *x* = 1.9–3.4. Table S1 summarizes the reaction parameters and resulting composition, size, and size distribution of Cu_*x*_SbSe_4_ nanocrystals. Generally, the fraction of Cu and Sb in the nanocrystals is proportional to the amounts of their halides.

### Kinetics of the Cu_*x*_SbSe_4_ Synthesis

Our systematic study of precursor composition offers us insights into the kinetics of the reaction and enables us to derive a predictive relation between the ratio of precursors CuCl:SbCl_3_ to the ratio of Cu to Sb in the resulting nanocrystals.

Assuming a co-precipitation reaction, the amount of Cu and Sb in the nanocrystals relates to the initial concentrations of halides via rate law equations. The ratio between Cu and Sb, *x*, in Cu_*x*_SbSe_4_ nanocrystals is given by:
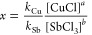
1where *a* and *b* are the partial reaction orders for CuCl and SbCl_3_ precipitation, *k*_Cu_ and *k*_Sb_ are reaction constants for halides, and [CuCl] and [SbCl_3_] are initial concentration of halides. By systematically plotting the experimentally determined *x* versus initial concentrations with different partial reaction orders for metal halides (Figure S2a,b) we find that a good linear fit is obtained assuming a partial order of 1 for SbCl_3_ and between 0.5 and 0.75 for CuCl (Figure S2c) These reaction orders are the number of metal halide molecules needed to precipitate 1 Se atom in the nanocrystal. Specifically, it suggests that the Sb intermediate complex is a selenochloride with SbCl_*x*_:Se stoichiometry of 1:1. We note that such selenohalides have been synthesized recently and applied for thin film solar cells.^35^ The fractional reaction order for CuCl implies a non-negligible opposite leaching reaction of Cu ions from the nanocrystal to the reaction solution. A similar process, usually promoted by surfactants, has been observed for CuInS_2_ nanocrystals.^36^ The proportionality constant of the best fit of eq 1 remains below 1 (, indicating that CuCl has a slower reactivity than SbCl_3_ (Figure 1e) at 130 °C. To summarize, this composition study provides us with the quantitative relation:  to enable us to predictively achieve Cu_*x*_SbSe_4_ nanocrystals of different stoichiometry (Figure S3).

### Mechanism of the Cu_*x*_SbSe_4_ Synthesis

To further understand the formation of Cu_*x*_SbSe_4_ nanocrystals, we perform a series of experiments where we systematically modify the amount of the Se precursor and reaction temperature (Figure 1f), while keeping the equimolar ratio of CuCl:SbCl_3_ = 3:1 and a growth time of 10 min. Increasing the amount of Se precursor results in higher Cu content (*x*) in the Cu_*x*_SbSe_4_ nanocrystals, which can be explained in the frame of hard and soft acids and bases (HSAB) concept. Excess selenide (soft base) promotes the reactivity of Cu ions (soft acid) relative to the hard Sb^(+5)^ acid.^15^ Consequently, the introduction of a slight excess of Se precursor (e.g., 40–60% extra Se) yields stoichiometric Cu_3_SbSe_4_ nanocrystals. In agreement with the HSAB explanation, highly Cu-rich nanocrystals form in case of extreme 2.5-fold Se excess (Figure 1f).

To explain this trend quantitatively, activation energies for each halide are calculated as follows. First, knowing the size and composition of Cu_*x*_SbSe_4_ nanocrystals for different temperatures and amounts of Se precursor, we calculate the number of Cu and Sb atoms, *N_i_*, in the nanocrystal with the following equation:
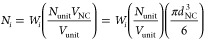
2where *W_i_* is the molar fraction of Cu or Sb, *V*_unit_ and *N*_unit_—the volume and total number of atoms in the famatinite unit cell, and *V*_NC_ and *d*_NC_—the volume and diameter of nanocrystals. Then, assuming negligible solid-state mass transfer (i.e., Ostwald ripening) at 10 min growth times, we extract atomic-specific activation energies directly from Arrhenius-type dependences for the temperature series with stoichiometric (100% Se) and excess (150% Se) amounts of Se precursor (Figure S4). According to the HSAB concept, CuCl is less stable than SbCl_3_, translating to a higher position along the potential energy axis of the reaction coordinate diagram (Figure 1g). For the stoichiometric amount of Se precursor, the activation energies of Cu and Sb are nearly the same (*E*_act, Cu_^100 % Se^ = 0.230 eV/atom and *E*_act, Sb_^100 % Se^ = 0.221 eV/atom), which results in notably higher energy of Cu intermediate species. We argue that this energy offset between Cu and Sb intermediates is the reason for Cu-deficient Cu_*x*_SbSe_4_ products when a stoichiometric amount of Se is used. The introduction of a Se excess lowers activation energy for CuCl (*E*_act, Cu_^150 % Se^ = 0.150 eV/atom), while simultaneously hindering the conversion of SbCl_3_ (*E*_act, Sb_^150 % Se^ = 0.297 eV/atom). Consequently, the energies of Cu and Sb intermediates become equal (Figure 1g), enabling stoichiometric Cu_3_SbSe_4_ nanocrystal products (Figure 1f). This quantitative explanation agrees with the HSAB concept and conclusions from the composition series (Figure 1e), suggesting that Cu and Sb intermediate species involve bonding to Se (i.e., soft base),^15^ as illustrated in Figure 1g. To sum up, our results exemplify how reactivities of cations can be balanced through the excess concentration of the anion precursor, highlighting a convenient and generalizable, yet often underestimated strategy for the composition control of ternary nanocrystals.

### Characterization of Cu_*x*_SbSe_4_ and Cu_3_SbSe_4_ Nanocrystals

**Figure 2:**
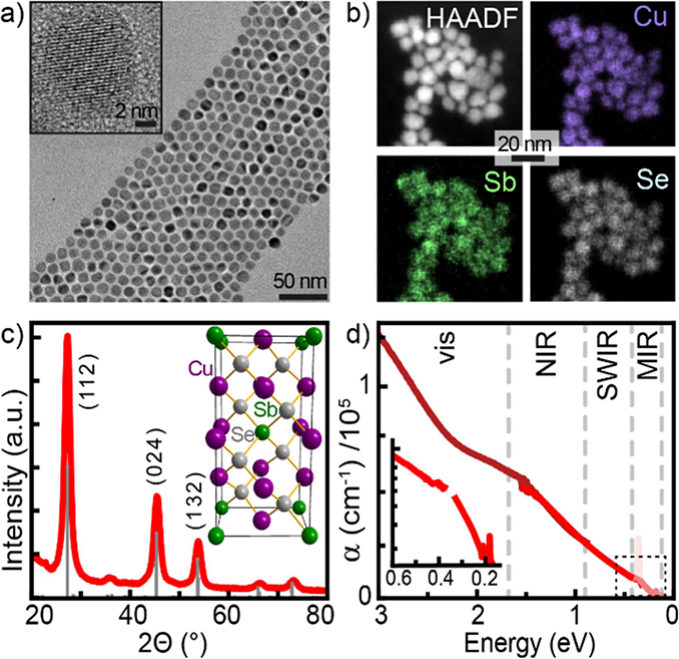
(a) TEM of stoichiometric Cu_3_SbSe_4_ nanocrystals. Inset shows high-resolution TEM of a single Cu_3_SbSe_4_ nanocrystal. (b) STEM/EDX composition map of Cu_3_SbSe_4_ nanocrystals. (c) XRD pattern of nanocrystals (red) and reference pattern of famatinite Cu_3_SbSe_4_ (gray). The inset shows the famatinite unit cell. (d) Absorption spectrum of Cu_3_SbSe_4_ nanocrystals. The inset shows the magnified plot range in logarithmic scale marked with dashed rectangle.

Electron microscopy of Cu_3_SbSe_4_ nanocrystals reveals excellent structural characteristics of Cu_3_SbSe_4_ nanocrystals, such as narrow size distribution below 10% (diameter is 10.7 ± 0.8 nm, Figure 2a), high crystallinity (Figure 2a, inset), and structural homogeneity of Cu, Sb, and Se within each nanocrystal and across the batch (Figure 2b). From the X-ray diffraction pattern of nanocrystals, we identify a famatinite structure, as expected for the bulk Cu_3_SbSe_4_ material (Figure 2c). The inset of Figure 2c shows a unit cell of the famatinite lattice, comprising a zinc blende superstructure with tetrahedral Se coordination for each cation. Moreover, stoichiometric Cu_3_SbSe_4_ nanocrystals show a narrow band gap and particularly high absorption coefficients in the visible and infrared ranges with an absorption onset of approx. 0.2 eV (Figure 2d).^12,13^

We also characterize the nanocrystals of different compositions obtained by varying the cation precursor ratio. Samples with varying compositions are similar in size and shape and have narrow size distributions (Figure S5). Furthermore, XRD patterns prove a famatinite structure for all investigated compositions (Figure S6 and Table S2), while STEM EDX maps indicate ternary Cu–Sb–Se composition of the nanoparticles for even the most Cu-deficient Cu_*x*_SbSe_4_ batch (*x* = 1.9, Figure S7). However, a closer look on STEM EDX linescans of Cu_*x*_SbSe_4_ nanocrystals with *x* = 1.9 reveals the presence of phase segregation within some nanocrystals (Figure S8), indicating the limit of the Cu_3_SbSe_4_–Cu_*x*_SbSe_4_ solid solution.

**Figure 3:**
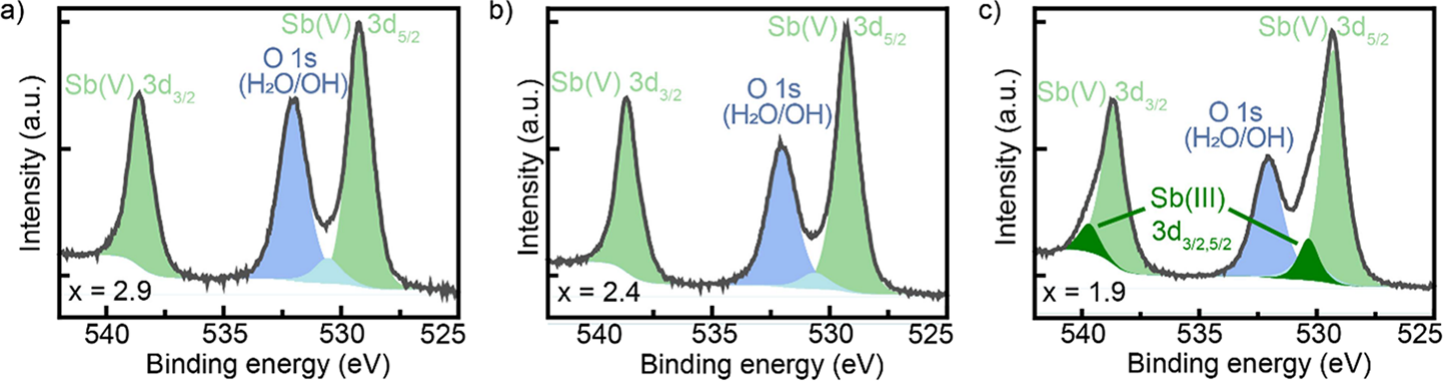
XPS spectra of the Sb 3*d* region for Cu_*x*_SbSe_4_ nanocrystals with (a) *x* = 2.9, (b) *x* = 2.4, and (c) *x* = 1.9.

To gain further insights into the solid solution, we perform XPS measurements of Cu_*x*_SbSe_4_ nanocrystals with *x* = 2.9, 2.4, and 1.9 (marked as open circles in Figure 1d,e). All samples contain Cu^(+1)^ and Se^(−2)^ species as sole oxidation states for these elements (Figures S9), in accordance with the previous literature.^14^ Auger peaks show no signs of metallic or oxidized atomic species (Figure S10). Sb 3*d* peaks occur at similar binding energies as the O 1*s* peak, where contributions from metal hydroxide and water adsorbed on the surface can be distinguished (Figure 3a–c). Nevertheless, for Cu_*x*_SbSe_4_ nanocrystals with *x* = 1.9, additional peaks corresponding to Sb^(+3)^ 3*d*_3/2_ and 3*d*_5/2_ can be detected, while for samples with *x* = 2.9 and *x* = 2.4, only peaks from Sb^(+5)^ are present. We therefore conclude that the famatinite structure is maintained for Cu_*x*_SbSe_4_ nanoparticles at least from *x* ≥ 2.4, while for more Cu-deficient nanocrystals, a nanoscopic secondary phase containing Sb^(+3)^ is formed within Cu_*x*_SbSe_4_ nanocrystals (Figure S8), possibly through the polytypism phenomenon at the nanoscale.^37^

In the bulk, slightly non-stoichiometric and homogeneous Cu_3_SbSe_4_ samples contain no more than 1% Cu deficiency,^38^ which is assigned to the formation of Cu vacancies.^21,27^ Larger non-stoichiometry leads to the formation of secondary phases, such as CuSe, Sb_2_Se_3_, or CuSbSe_2_.^39,40^ In contrast, we find that Cu_3_SbSe_4_ nanocrystals tolerate at least 20% of Cu vacancies. The tolerance to Cu-vacancies in Cu_3_SbSe_4_ nanocrystals can be tied to the small crystal domain size in nanocrystals, where phase segregation of secondary phases is likely to be less energetically favorable than the incorporation of additional point defects. This indicates nanoscale Cu_*x*_SbSe_4_ materials with previously unknown compositions.

### Electronic Structure Calculation for Cu_*x*_SbSe_4_ Nanocrystals

We turn to theoretical calculations to gain further insights into the electronic structure of the nanocrystals, their optical properties, and the impact of defect chemistry. The systems are relatively large, with a 9.5 nm diameter nanocrystal containing ∼20,000 atoms. To enable efficient atomistic computation of the electronic structure of many nanocrystals, with different sizes and defects (e.g., Cu vacancies), we use density functional theory (DFT)-parametrized tight-binding simulations because DFT calculations of such nanocrystals are computationally too intensive for the exploration of multiple samples.

**Figure 4:**
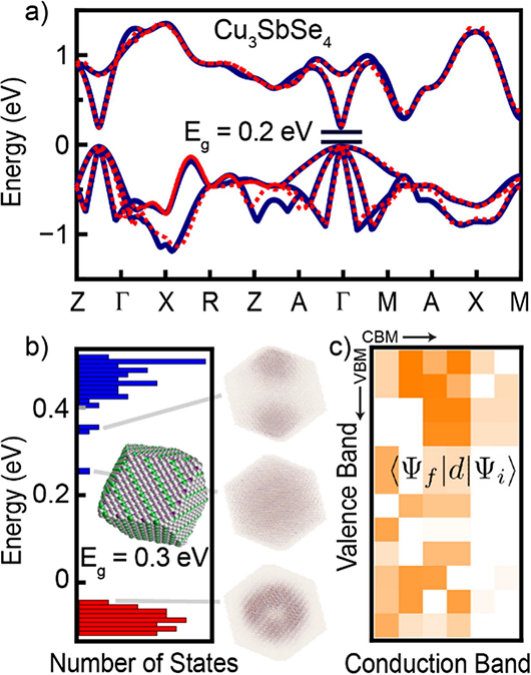
(a) Electronic band structure of bulk famatinite Cu_3_SbSe_4_, as obtained from the DFT (solid dark blue) and fitted tight-binding model (dotted red). (b) Energy distribution of the conduction and valence band states and selected electron densities for the Cu_3_SbSe_4_ nanocrystal from tight-binding calculations. The inset shows a nanocrystal with (110) facets and approx. 20,000 atoms. (c) Optical coupling between the first few valence (vertical) and conduction (horizontal) band states, starting from the band edges (top left corner of the plot). Darker color indicates stronger optical coupling (logarithmic color scale).

We perform bulk DFT calculations on the famatinite structure. Small band gap semiconductors typically require computationally expensive hybrid functionals to accurately represent the band gap. By adapting the previously developed method for a 144-atom unit cell,^3^ we achieve a prediction of the bulk band gap consistent with the literature for a unit cell reduced to 16 atoms (Cu_6_Sb_2_Se_8_) to facilitate parameter fitting. (Figure 4a). We find tight-binding parameters by fitting to the bulk band structure.^31^Table S3 summarizes the details of the parameter fitting procedure.

We then artificially generate Cu_3_SbSe_4_ nanocrystals by cutting an infinite periodic structure along (110) facets and calculate the electronic structure with the tight-binding code OMEN (Figure 4b). Valence and conduction bands each have distinct atomic and orbital participations with the valence band predominantly formed by cationic states (>90% Cu and Sb) and the conduction band mostly by Se states (>80%) (Figure S11). We define the bandgap as the energy difference between the highest valence band state and the lower conduction band state. The first four states of the conduction band structure consist of a single, *s*-type state, and three *p*-type triplet states (Figure 4b). Strong optical coupling with the *d*-type valence band wavefunctions (Figures 4c and S12) implies that absorption will primarily occur from the top of the valence band to the triplet states in the conduction band. These characteristics are similar to those of I–III–VI nanocrystals, previously studied with tight-binding simulations.^41^

A 9.5 nm nanocrystal has a band gap of 0.3 eV and a main absorption feature from the valence band to the triplet states at 0.38 eV (Figure S13). Reducing the nanocrystal size from 9.5 to 7.5 nm increases the band gap from 0.3 to 0.35 eV, and the 10% size variation in nanocrystals observed experimentally could lead up to a 50 meV spread in band gap energies (Figure S14a).

**Figure 5:**
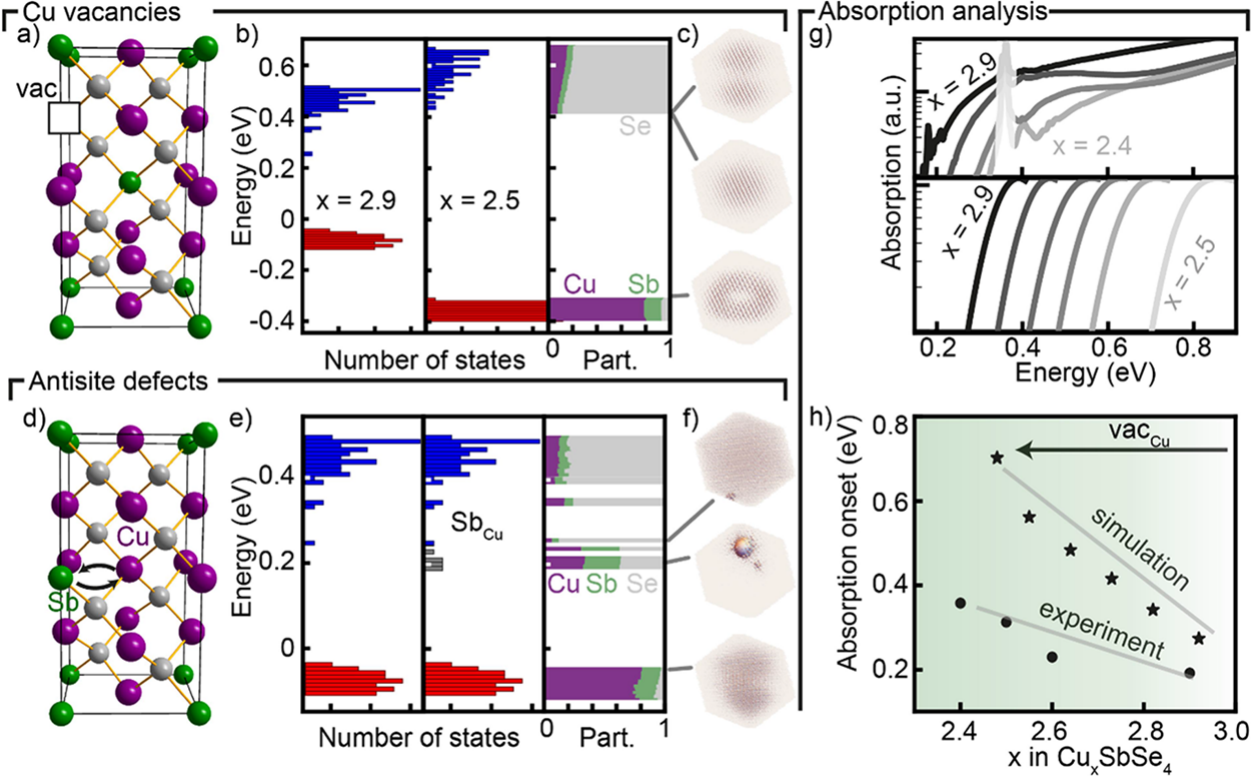
(a) Single Cu vacancy in the famatinite unit cell. (b) Eigenmodes of stoichiometric Cu_3_SbSe_4_ (left), Cu-deficient Cu_2.5_SbSe_4_ nanocrystals (center), and atomic participation of Cu_2.5_SbSe_4_ nanocrystals showing unperturbed valence and conduction band states (right). (c) Selected electron densities of Cu_2.5_SbSe_4_ nanocrystal. (d) Stoichiometric antisite defect in the famatinite unit cell. (e) Eigenmodes of stoichiometric Cu_3_SbSe_4_ (left), Cu_3_SbSe_4_ with 7 antisite defect pairs (center), and atomic participation (right) of the Cu_3_SbSe_4_ nanocrystal with antisite defects showing a clear difference between the valence band (mostly Cu and Sb), conduction band (mostly Se), and defect states (mixed). (f) Selected electron densities of the Cu_3_SbSe_4_ nanocrystal with antisite defects, showing localized defect states. (g) Experimental (top) and simulated (bottom) absorption of Cu_*x*_SbSe_4_ nanocrystals, plotted in logarithmic scale. (g) Comparison of absorption onset from experiment (circles) and simulated (stars) Cu_*x*_SbSe_4_ nanocrystals.

To achieve compositions with different amounts of Cu vacancies, we remove Cu atoms from the artificially generated nanocrystal (Figure 5a). The passivation of dangling bonds within the nanocrystal represents an electron for charge compensation, thus implying a neutral vacancy. The case of 1 out of every 6 Cu atoms being removed (*x* = 2.5) is shown in Figure 5b. The introduction of vacancies separates the valence and the conduction band states leading to an increase of the band gap similar to observations in Cu–In–Se-ordered vacancy compounds.^42^ Comparing *x* = 2.9 and *x* = 2.5, the band gap increases from 0.3 to 0.77 eV (Figures 5b and S15). Cu vacancy corresponds to a missing positive charge around Se atoms that is partially compensated by neighboring Cu and Sb cations. This increases the energy of the conduction band states and reduces the energy of the valence band states. Vacancies do not impact the atomic and orbital participation in states (Figure 5b and S16), and electron wavefunctions largely maintain their delocalized characteristics (Figure 5c and S17).

We next explore Cu_Sb_ and Sb_Cu_ antisite defects, which is another type of structural disorder found often in multicomponent semiconductors. Bulk DFT calculations predict a higher formation energy of antisite defects (3 eV in Cu_3_SbS_4_^43^) compared to cation vacancies (0.65 eV in Cu_3_SbSe_4_),^26^ so we expect a relatively low concentration of Cu_Sb_ and Sb_Cu_. In our simulations, we introduce an antisite defect pair by exchanging Cu and Sb cations leading to the creation of both a Cu_Sb_ and a Sb_Cu_ defect (Figure 5d). This leads to the appearance of a single defect state in the band gap at different energies depending on its position in the nanocrystal and on the presence of adjacent Cu vacancies (Figures S18 and S19). Interestingly, only Sb_Cu_ creates mid-gap states (Figures S20 and S21).

For the case of stoichiometric Cu_3_SbSe_4_ nanocrystals with 7 randomly introduced defect pairs (0.5% antisite defects, Figure 5e), the Sb_Cu_ antisite defects each form one mid-gap state, which appear mostly a few tens of meV below the conduction band edge (Figures 5e and S22).

Sb_Cu_ antisite defect states lead to (Cu, Cu, Sb, and Sb) tetrahedra around Se atoms with 12 instead of 8 positive charges. This heavily affects neighboring atoms, resulting in mixed atomic and orbital participation of the localized defect state (Figures 5e and S23).

The wavefunction of defect states is localized, while those of the lowest conduction and highest valence states remain largely unaffected (Figure 5f). Similar results are found for introducing antisite defect pairs in off-stoichiometric Cu_*x*_SbSe_4_ (Figure S24).

### Reconciling Experiment and Computation

We perform composition-dependent FTIR spectroscopy to obtain the absorption spectra of Cu_*x*_SbSe_4_ nanocrystals. As the number of Cu vacancies increases, the absorption onset shifts to higher energies (Figure 5g, top and Figure S25). We note that the samples with *x* ≤ 2.1 exhibit a different absorption profile, likely related to the appearance of a secondary phase containing Sb^(+3)^ (Figure S26). The simulated absorption of perfectly ordered Cu_*x*_SbSe_4_ (*x* = 2.5–2.9, Figures S14b and S15) nanocrystals is shown in Figure 5g (bottom) and shows an increase in the absorption onset as expected from band structure calculations. While the same trend in absorption onset is visible, the computations predict an approximately 50 meV larger absorption onset than that measured experimentally, and the difference grows with increasing vacancy concentration (Figure 5h). This could be due to antisite defects, which lead to finite absorption within the band gap even at very low concentrations (0.5% Sb in simulated nanocrystals, Figures S23 and S24); however, the facts that the offset is consistent across compositions and that the experimental absorption onset is distinct suggest that the offset may be due to a systematic difference between experiment and simulation. For example, increasing vacancy concentrations typically lead to changes in unit cell size and lattice rearrangements.^44^ In addition, the offset may be due to the fact that in experiment, the nanocrystals are close-packed in the thin-film and have a finite size dispersion while computation was done on individual nanocrystals in vacuum without excitons, surface distortions, ligands, or nearest neighbors.^45^

## Conclusions

In this paper, we study the synthesis of Cu_*x*_SbSe_4_ nanocrystals and the impact of composition and defects on their electronic structure and optical properties. We develop a colloidal synthesis recipe to achieve size-uniform, non-stoichiometric Cu_*x*_SbSe_4_, and stoichiometric Cu_3_SbSe_4_ nanocrystals. We find that Cu_*x*_SbSe_4_ nanocrystals tolerate a much higher concentration of Cu-vacancies (at least *x* = 2.4–3.0), compared to the bulk Cu_*x*_SbSe_4_ phase (where *x* = 2.97–3.00).

We calculate DFT parametrization for Cu_3_SbSe_4_ and use tight-binding simulations to reveal the impact of Cu-vacancies and antisite defects on the optical properties of Cu_*x*_SbSe_4_ nanocrystals. We find that the band gap increases as the amount of Cu vacancies increases, a trend which we confirm by infrared spectroscopy. Sb_Cu_ antisite defects lead to mid-gap states, which can couple to the band state; however, there is no clear evidence of their presence from optical absorption measurements.

The control and understanding over the chemical synthesis developed here as well as insights into the electronic structure provides the toolbox for future studies on nanocrystalline Cu_*x*_SbSe_4_ for optoelectronic or thermoelectric applications. For example, electrochemical analysis or ultraviolet photoelectron spectroscopy can provide valuable information about band onset energies,^46^ or the choice of appropriate surface ligands^14^ can accurately tune the conductivity of nanocrystal thin films, thus enabling a targeted device design.

## Supporting Information Available

Detailed synthesis parameters; additional parameter plane maps; extended XPS and absorption data; tight-binding parameters; and additional computational results (PDF)
