# Supplementary material for: Synthesis and Electronic Structure of Mid-Infrared Absorbing Cu3SbSe4 and CuxSbSe4 Nanocrystals
Source: Chem Mater. 2023 Aug 9;35(16):6323–31. doi: 10.1021/acs.chemmater.3c00911 (PMC10448677; doi:10.1021/acs.chemmater.3c00911)
Supplement: Supplementary file 1 — cm3c00911_si_001.pdf [file cm3c00911_si_001.pdf]

Supporting Information for

Synthesis and Electronic Structure of mid-IR Absorbing Cu_3SbSe_4 and Cu_xSbSe_4 Nanocrystals

Annina Moser,[1,‡] Olesya Yarema,[1,‡] Gregorio Garcia,[2,†] Mathieu Luisier,[3] Filippo Longo,[4] Emanuel Billeter,[5] Andreas Borgschulte,[4,6] Maksym Yarema,[1] and Vanessa Wood[1,*]

[1] Institute for Electronics, Department of Information Technology and Electrical Engineering, ETH Zurich, Gloriastrasse 35, CH-8092 Zurich, Switzerland

[2] Departamento de Tecnología Fotónica y Bioingeniería & Instituto de Energía Solar, ETSI Telecomunicación, Universidad Politécnica de Madrid, Ciudad Universitaria, s/n ES-20840 Madrid, Spain

[3] Institute for Integrated Systems, Department of Information Technology and Electrical Engineering, ETH Zurich, Gloriastrasse 35, CH-8092 Zurich, Switzerland

[4] Laboratory for Advanced Analytical Technologies, Empa, Überlandstrasse 129, CH-8600 Dübendorf, Switzerland

[5] Department of Physics, Danmarks Tekniske Universitet, Fysikvej, Building 312, 2800 Kgs. Lyngby Denmark

[6] Department of Chemistry, University of Zurich, Winterthurerstrasse 190, CH-8057 Zürich, Switzerland

* vwood@ethz.ch

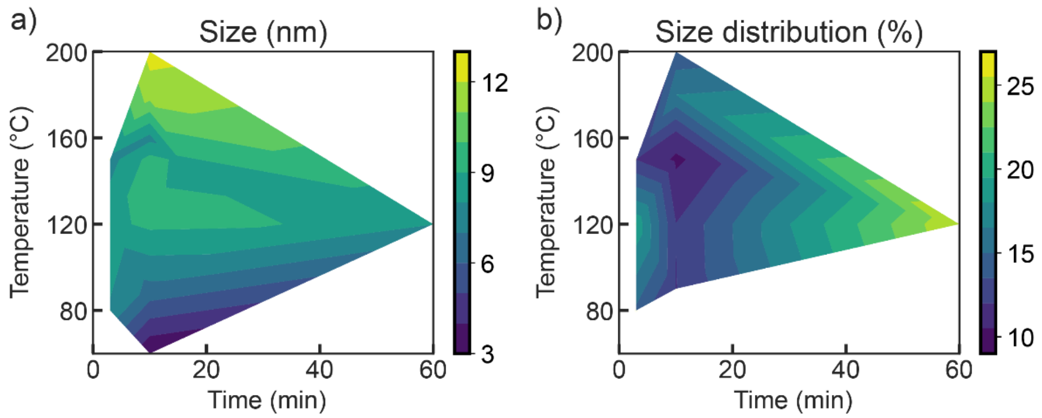

Figure S1. a) Cu_xSbSe_4 nanocrystal size increases with temperature and is independent of reaction time. b) Nanocrystal size distribution is minimal at 10 minutes reaction time for all temperatures. Due to the significantly smaller size at 70°C reaction temperature, the size distribution is not comparable and thus not plotted in this graph

Table S1. Reaction parameters and characterization of Cu_xSbSe_4 nanocrystal composition series used to determine the reaction order (**Figure 1d**). Shaded samples are characterized in more detail.

**Precursor ratio**		**SEM/EDX**				**TEM**
**CuCl (mmol)**	**$SbCl_3$ (mmol)**	**at% Cu**	**at% Sb**	**at% Se**	**Cu/Sb**	**Size (nm)**
0.625	0.275	33	17	50	1.9	9.75 ± 1.71
0.55	0.25	34	17	49	2	10.41 ± 1.77
0.65	0.25	34	16	50	2.1	9.74 ± 1.60
0.75	0.25	36	15	49	2.4	9.30 ± 1.68
0.75	0.275	40	16	44	2.5	9.93 ± 2.83
0.85	0.25	35	14	51	2.5	12.10 ± 3.20
0.75	0.225	39	15	41	2.6	7.67 ± 1.14
0.875	0.225	40	14	46	2.9	8.50 ± 0.96
0.65	0.2	36	12	52	3	8.20 ± 1.33
0.75	0.2	41	13	46	3.2	8.40 ± 0.96
0.85	0.2	41	12	47	3.4	10.60 ± 1.88
0.85	0.2	42	12	46	3.5	7.98 ± 0.99

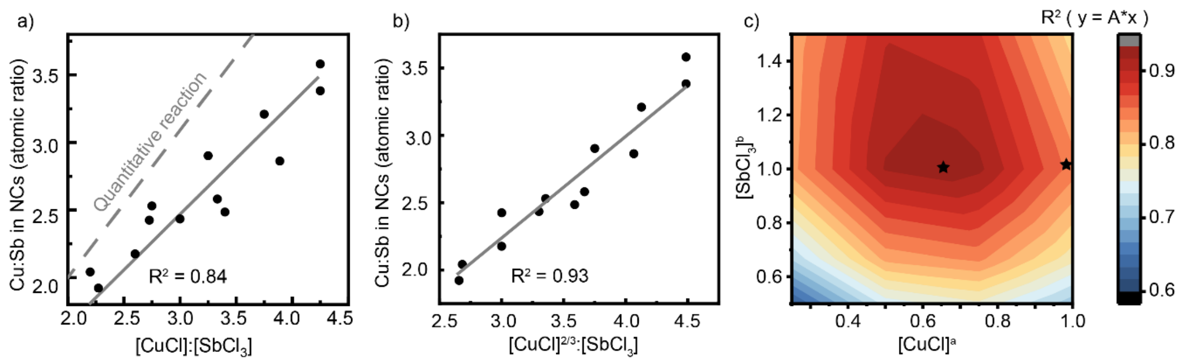

Figure S2. a) Relative reactivity plot of the Cu_xSbSe_4 nanocrystal synthesis. The reaction order fit of [CuCl]:[$SbCl_3$] fails to accurately represent the observed cation content in the synthesized nanocrystals. b) A reaction order of x ∝ [CuCl] $^{2/3}$:[$SbCl_3$] exhibits the best representation of content of single cation species as well as the Cu/Sb ratio. c) Map of R^2 goodness of the y = A×*x* fit for different reaction orders for CuCl and $SbCl_3$. The displayed fits of a) and b) are marked with stars.

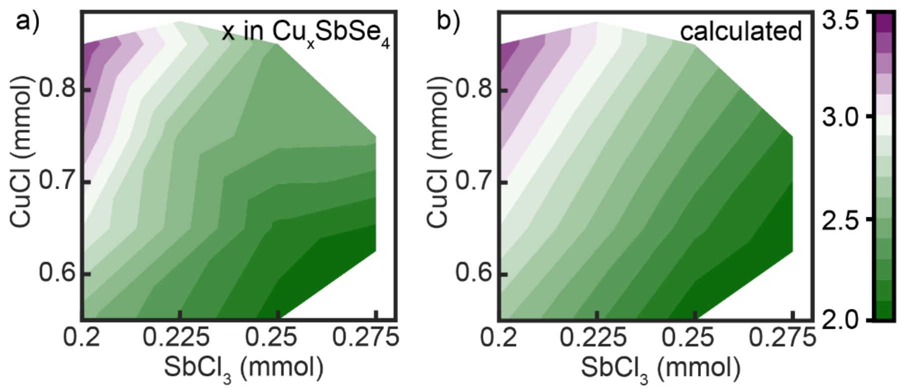

Figure S3. a) Experimental and b) calculated composition maps according to the [CuCl] $^{2/3}$:[$SbCl_3$] fit (**Figure S2b**) show an excellent agreement.

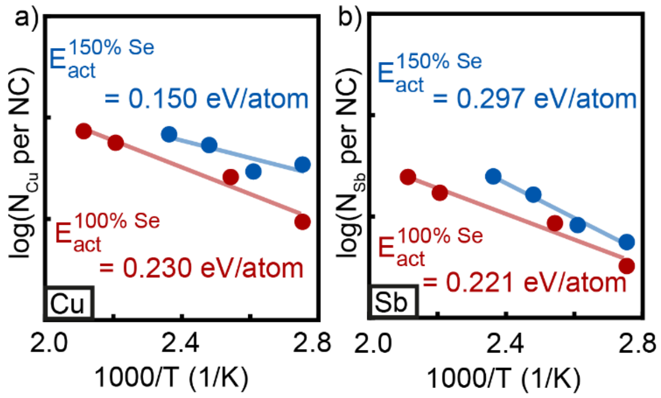

Figure S4. a) Arrhenius-type fit of the number of Cu atoms, and b) number of Sb atoms in the nanocrystal. Data for a stoichiometric amount of Se precursor (100% Se) are in red and for the 1.5-fold excess Se (150% Se) are in blue. Activation energies are extracted from fitted slopes.

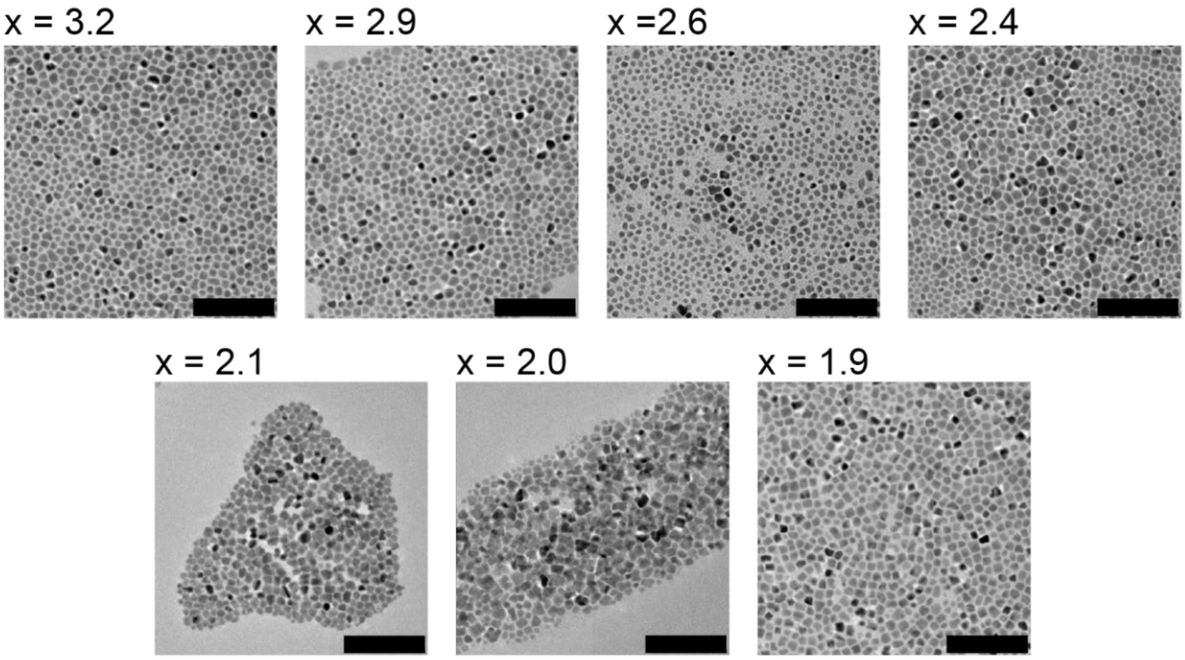

Figure S5. TEM images of Cu_xSbSe_4 composition series with various x. All samples show close to uniform particles with increasing deviation for Sb-rich particles. The scale bar corresponds to 100nm.

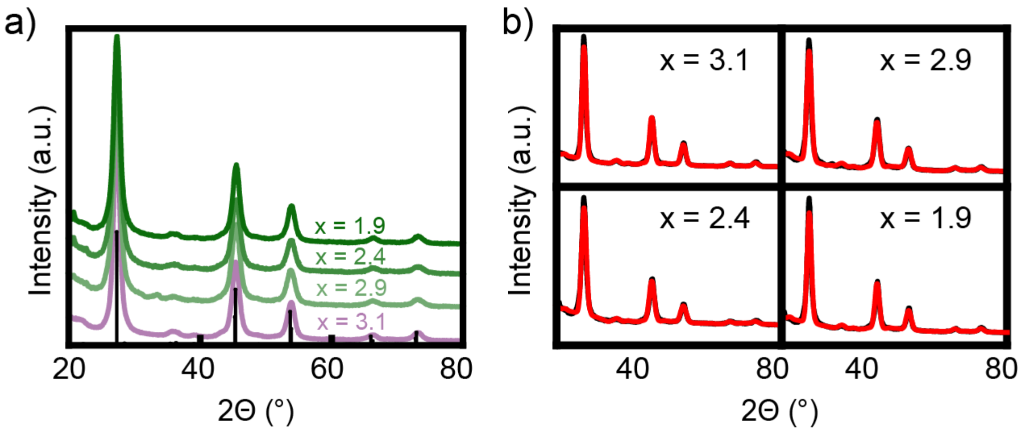

Figure S6. a) Composition-dependent XRD and b) Rietveld refinement fit for various x in Cu_xSbSe_4 nanocrystals.

Table S2. Unit cell parameters of Rietveld refinement from figure S8b. The unit cell volume appears to decrease marginally with increasing Sb-content.

Material	a (Å)	c (Å)	Unit cell volume (Å^3)	Bragg R-factor	Chi2
x = 3.1	5.66622	11.25822	361.457	6.96	34.2
x = 2.9	5.66642	11.26736	361.776	11.4	24.8
x = 2.4	5.66425	11.26429	361.4004	6.37	13.2
x = 1.9	5.66156	11.24740	360.5159	9.57	30.9

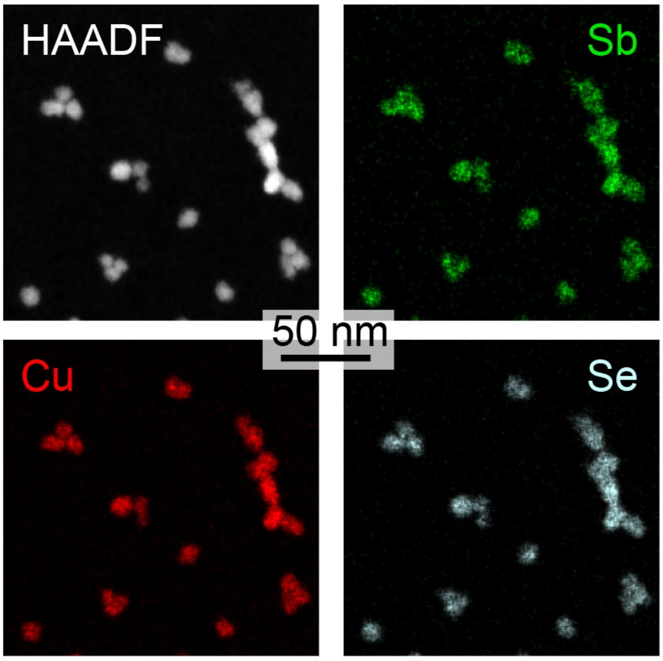

Figure S7. STEM/EDX composition maps of Cu_xSbSe_4 with x = 1.9. No secondary phase is visible with the given resolution.

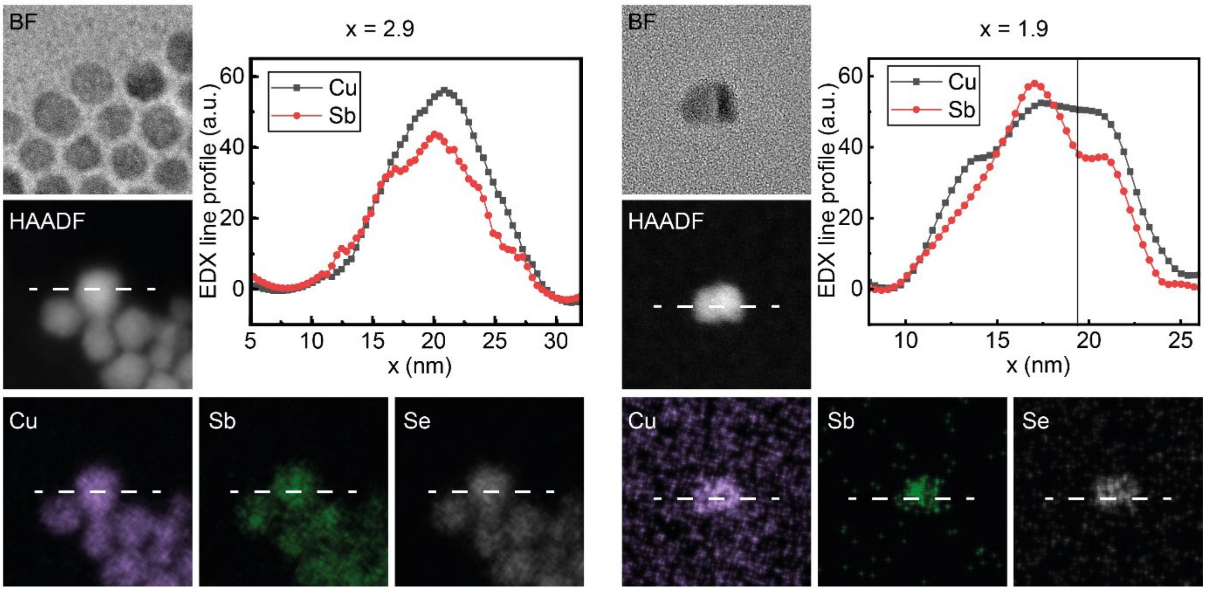

Figure S8. STEM/EDX maps of stoichiometric (x=2.9) and non-stoichiometric (x=1.9) Cu_xSbSe_4 nanocrystals with corresponding elemental line profiles. Bright field image for the sample with x = 2.9 is from a different grid location of the same sample. This result indicates the presence of secondary phases in highly Cu-deficient nanocrystals, however, the achievable spatial resolution is not sufficient for quantitative composition results.

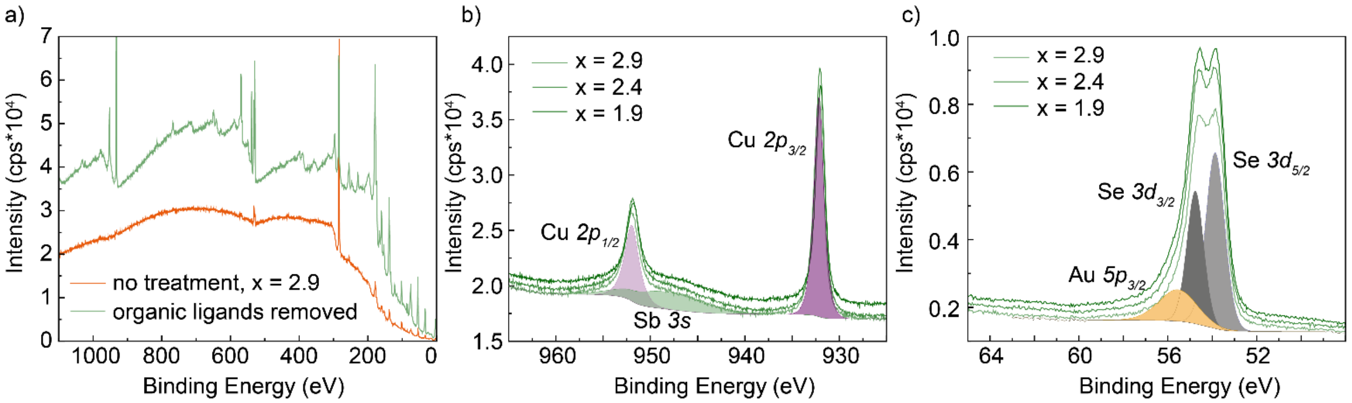

Figure S9. a) XPS survey spectra of stoichiometric Cu_3SbSe_4 nanocrystals with (orange) and without (green) organic ligands. The presence of an organic layer on top of the nanoparticle film reduces the XPS signal. Inelastic scattering at the interface of organic and inorganic matter leads to weaker photoelectron peaks compared to the background.[1] XPS detail scans of b) Cu *2p* and c) Se *3d* of Cu_xSbSe_4 with various x show no sign of secondary oxidation states. Exemplary line fittings are shown for stoichiometric Cu_3SbSe_4 nanocrystals (x=2.9).

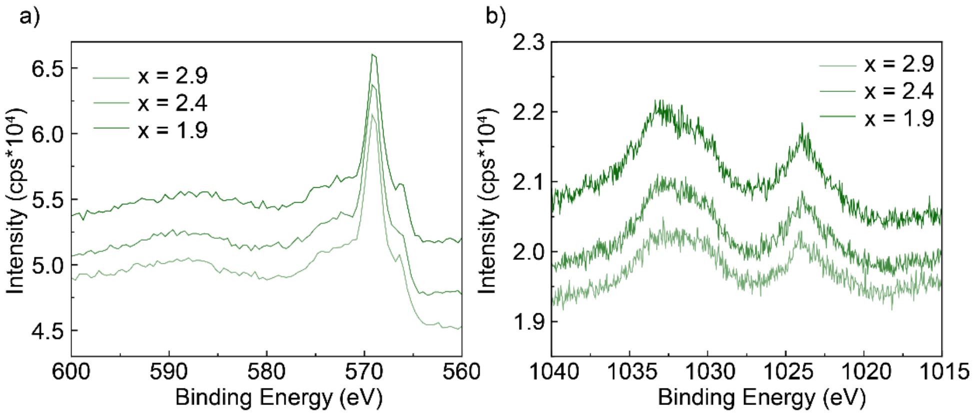

Figure S10. Auger peaks extracted from the XPS survey spectrum for Cu_xSbSe_4 nanocrystals with various x. No significant difference between compositions is found. a) Auger parameter peaks of Cu are very similar to CuI and Cu_2S but very different to the ones of CuO and other Cu^{2+} species. b) Auger parameter peaks of Sb are very close to Sb_2S_5 and Sb_2S_3, but not close to Sb_2O_3 or Sb^0. This indicates that the nanocrystal surface atoms are not oxidized.

[1] Baer, D. R.; Engelhard, M. H. XPS Analysis of Nanostructured Materials and Biological Surfaces. *J. Electron Spectros. Relat. Phenomena* **2010**, 178–179 (C), 415–432. https://doi.org/10.1016/J.ELSPEC.2009.09.003.

Table S3. Fitted tight-binding parameters for bulk famatinite Cu_3SbSe_4 unit cell containing 16 atoms. For Cu, sp^3d^5 orbital are chosen, while for Sb and Se, sp^3s^* are used. The parameter fitting is not fully relaxed, since the fitting algorithm restricts the interaction parameters V to have the correct sign and a limited magnitude.

Es_{Cu}	20.5548	**Cu-Se**		**Se-Cu**		**Sb-Se**		**Se-Sb**	
Ep_{Cu}	-13.7295	V_{ss}	-2.2689	V_{sp}	0.1077	V_{ss}	-5.4255	V_{ss^*}	-0.2131
Ed_{Cu}	-26.0478	V_{sp}	9.9963	V_{sd}	-10.0000	V_{sp}	1.3640	V_{sp}	9.9996
Es_{Sb}	-9.6870	V_{pps}	3.1681	V_{s^*p}	1.7650	V_{ss^*}	-3.5663	V_{ps^*}	8.5374
Ep_{Sb}	-8.3504	V_{ppp}	-2.5704	V_{s^*d}	-5.4925	V_{ps^*}	7.9776	$V_{s^*s^*}$	-0.9656
Es^*_{Sb}	12.0579	V_{ss^*}	-2.4065	V_{pds}	-0.8203	V_{pps}	7.5187		
Es_{Se}	-17.3561			V_{pdp}	2.9376	V_{ppp}	-2.5431		
Ep_{Se}	3.1559								
Es^*_{Se}	-11.4224								

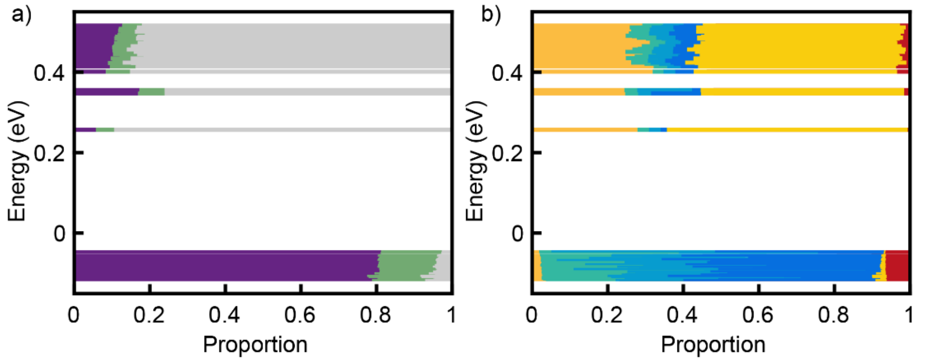

Figure S11. a) Atomic (purple – Cu, green – Sb, grey - Se) and b) orbital (dark yellow – s, blues – p, yellow – s*, red – d) participation for a perfectly ordered, stoichiometric Cu_3SbSe_4 nanocrystal. Clear differences permit unique identification of bands in the case of incorporated defects.

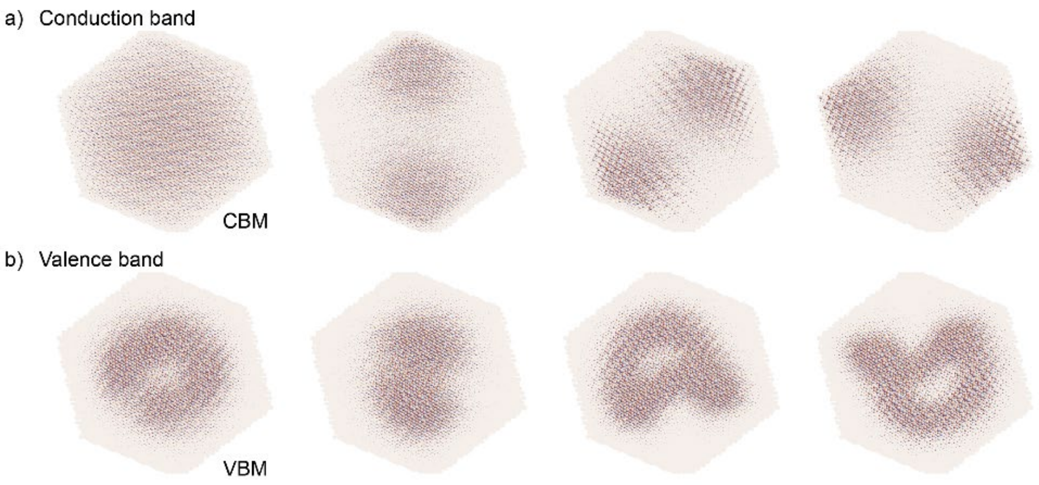

Figure S12. Electron densities of the first four wavefunctions of the a) conduction and b) valence band of a perfectly ordered, stoichiometric Cu_3SbSe_4 nanocrystal.

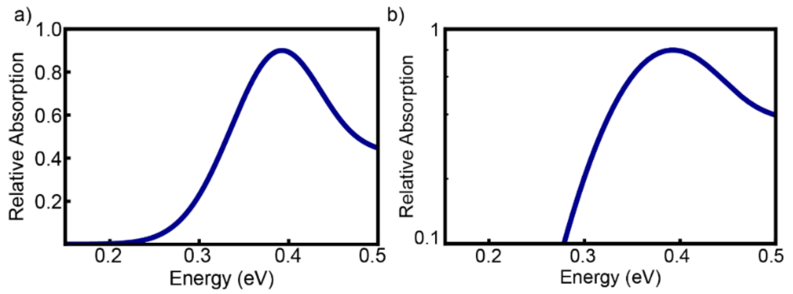

Figure S13. a) Calculated absorption for perfectly ordered, stoichiometric Cu_3SbSe_4 nanocrystal on a linear and b) logarithmic scale. A broadening of 50 meV is employed for absorption simulation representing the band gap variation according to experimentally observed size distribution of 10%. The peak at 0.4 eV corresponds to a particularly strong band-to-band absorption and is likely to be averaged out to a featureless spectrum as experimentally observed due to nanocrystal structure variation of the measured ensemble.

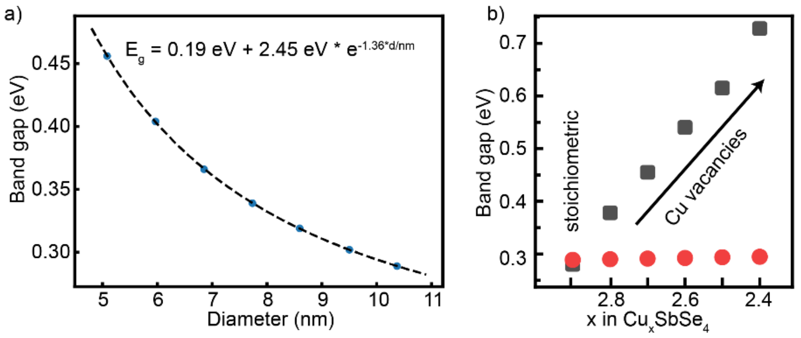

Figure S14. a) Size series of stoichiometric Cu_3SbSe_4 nanocrystals. The band gap tends towards 0.19 eV, which is slightly below the bulk band gap of 0.2 eV. b) Band gaps of stoichiometric and vacancy-containing structures (black squares) change significantly more compared to the theoretical band gap change due to the reduced number of atoms (red circles) according to the formula derived from the size series in a).

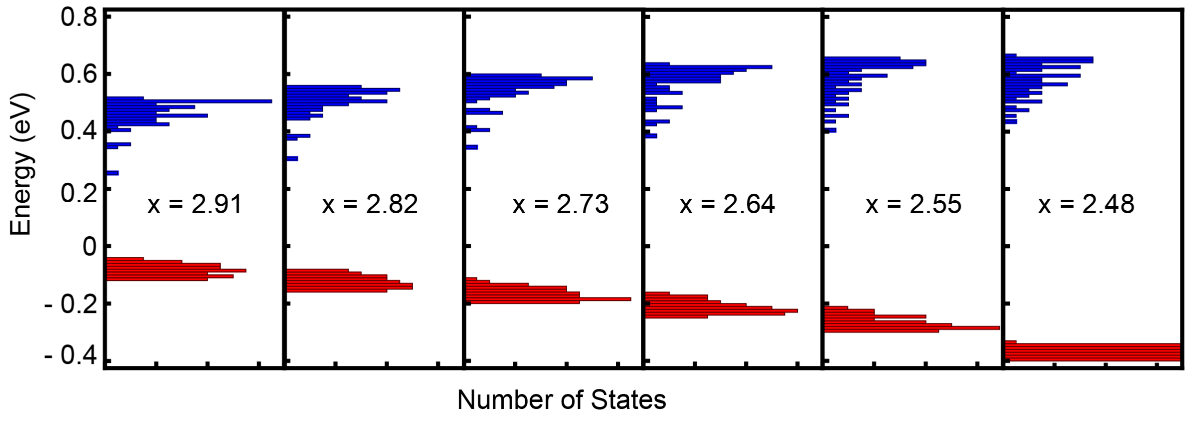

Figure S15. Density of states for simulated Cu_xSbSe_4 nanocrystals with increasing amounts of vacancies between stoichiometric nanocrystals (x=2.91) and Cu_xSbSe_4 with x = 2.48. The conduction band edge is increased, while the valence band edge energy is reduced, increasing the band gap from 0.3 eV to 0.77 eV. A primary change in the valence band suggests that charge compensation is performed mostly by cations.

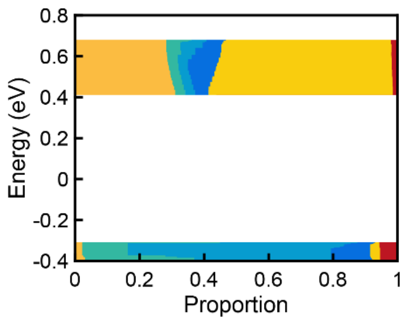

Figure S16. Orbital participation (dark yellow – s, blues – p, yellow – s*, red – d) for Cu_xSbSe_4 with x = 2.48. The character of the bands is largely unchanged compared to the structure without vacancies (see **Figure S11**).

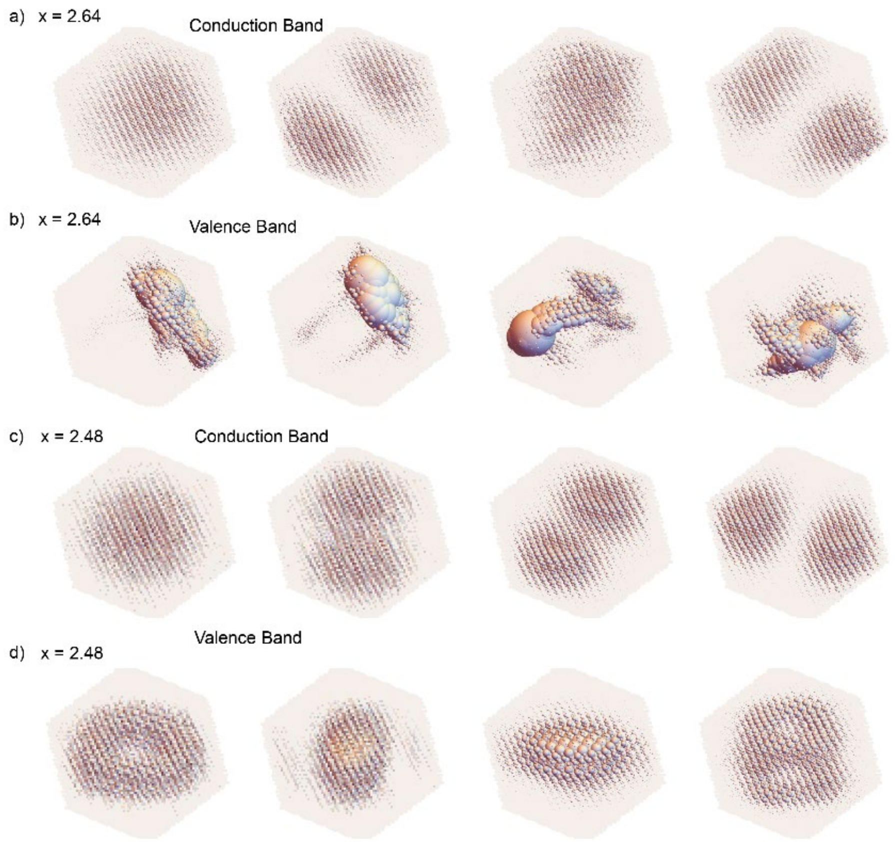

Figure S17. Electron densities of the first four wavefunctions of the a), c) conduction and b), d) valence band of two Cu_xSbSe_4 nanocrystals containing increasing amounts of vacancies. The periodic structure for x = 2.48 leads to delocalized valence band states, while the symmetry breaking in the structure with x = 2.64 due to a random arrangement of vacancies causes increased localization of valence band states.

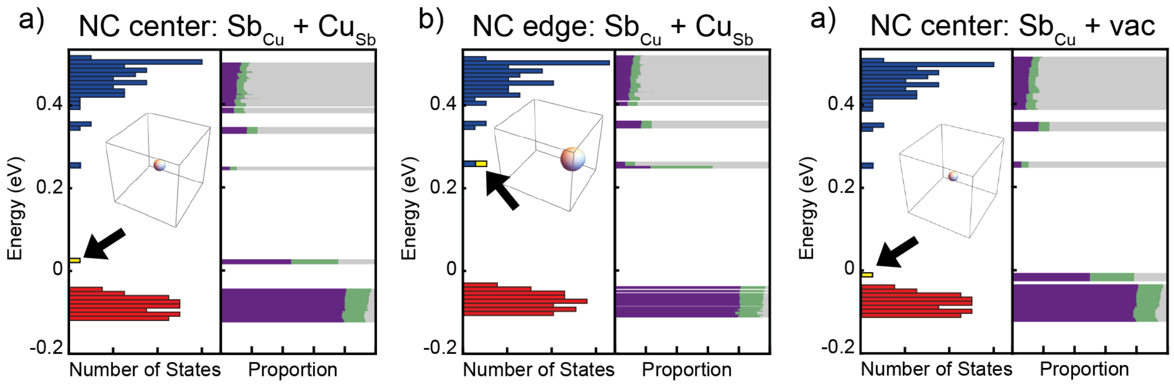

Figure S18. Density of states and atomic participation for simulated stoichiometric Cu_3SbSe_4 nanocrystals with one Sb_{Cu} + Cu_{Sb} antisite defect pair a) in the center, b) at the surface of the nanocrystal and c) one Sb_{Cu} + adjoining vacancy in the center with electron wavefunctions of defect states (yellow). While the defect energy level is unaffected by position within the core, the energy is shifted just below the conduction band edge for a defect at the surface. For a defect in close vicinity of a vacancy, the energy is shifted closer to the valence band edge.

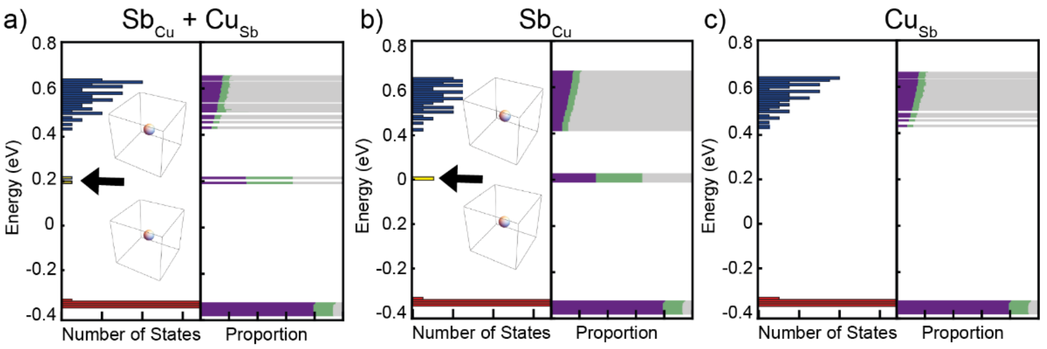

Figure S19. Density of states and atomic participation for simulated Cu_xSbSe_4 nanocrystals (x = 2.48) with a) one Sb_{Cu} + Cu_{Sb} antisite defect pair, b) one Sb_{Cu} defect and c) one Cu_{Sb} defect in the center of the nanocrystal with electron wavefunctions of defect states (yellow). The Sb_{Cu} defect creates two defect states in the presence of high vacancy densities, which is not related to the presence of a Cu_{Sb} defect.

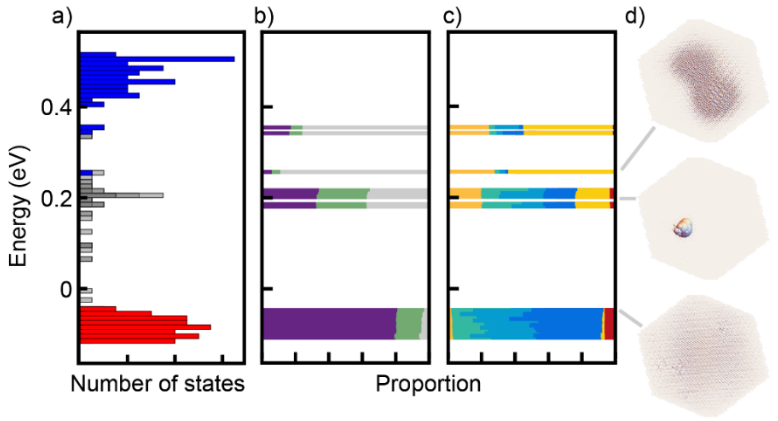

Figure S20. a) Density of states of a perfectly ordered stoichiometric nanocrystal (red/blue) and for 10 different random introductions of 7 Sb_{Cu} antisite defects per nanocrystal (light gray). b) Atomic and c) orbital participation, as well as d) selected wavefunctions show localized eigenmodes distinct from the valence and conduction bands.

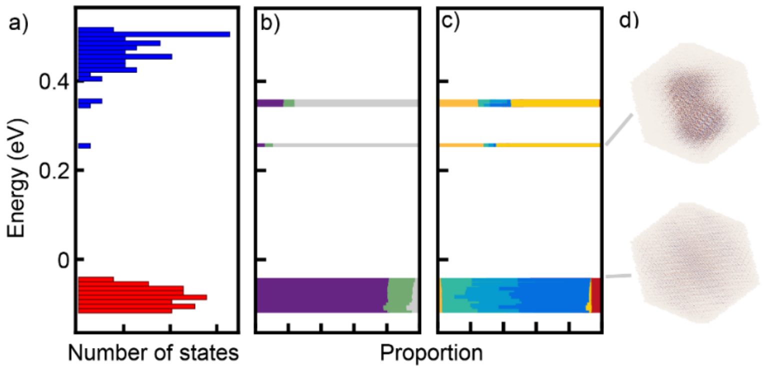

Figure S21. Density of states of a perfectly ordered stoichiometric nanocrystal (red/blue) and for 10 different random introductions of 7 Cu_{Sb} antisite defects per nanocrystal (no additional states are visible). b) Atomic and c) orbital participation shows no defect states; d) wavefunctions of valence band maximum and conduction band minimum are not perturbed. Defect states are most likely situated in the valence band with mostly Cu-character but cannot be distinguished from regular valence band states. Alternatively, defect states are significantly lowered in energy such that they do not appear in these calculations.

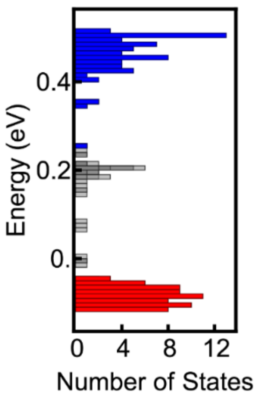

Figure S22. Density of states of a perfectly ordered stoichiometric Cu_3SbSe_4 nanocrystal (red/blue) and for 10 different random introductions of 7 stoichiometric antisite defect pairs per nanocrystal (light gray). Defect states are statistically more likely to appear close to the surface and may be in proximity of other defect states, which is why the most probable energy level differs from that of a single defect state (see **Figure S18**).

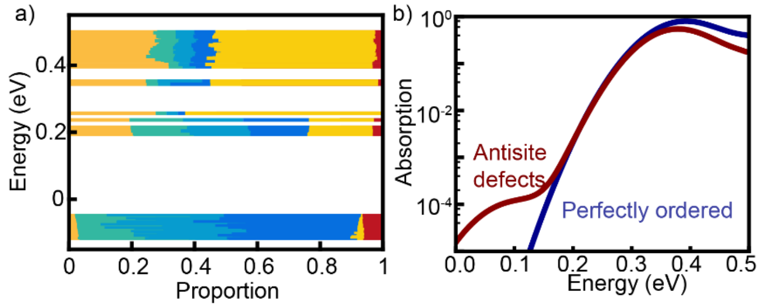

Figure S23. a) Orbital participation for a stoichiometric Cu_3SbSe_4 nanocrystal with antisite defects. The orbital participation of defect states is clearly distinct from valence and conduction band states. b) Simulated absorption for stoichiometric Cu_3SbSe_4 nanocrystal without (blue) and with (red) antisite defects. Finite sub-bandgap absorption is visible in the presence of antisite defects.

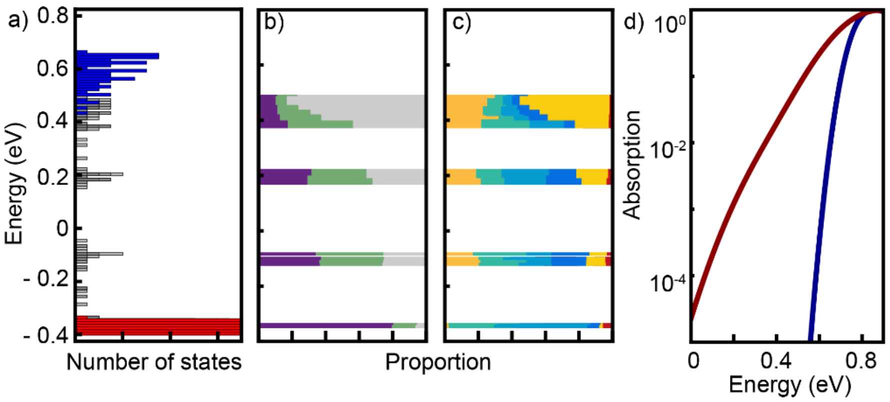

Figure S24. a) Density of states of perfectly ordered Cu_xSbSe_4 with x = 2.48 (red/blue) and for 7 different random introductions of 7 stoichiometric antisite defect pairs per nanocrystal (light gray). b) Atomic and c) orbital participation of single nanocrystal with vacancies and antisite defects. d) Calculated absorption for Cu_xSbSe_4 without (dark blue) and with (red) antisite defects. Due to the interaction of antisite defects with vacancies (see **Figure S18**), the impact of antisite defect states on the electronic structure is increased.

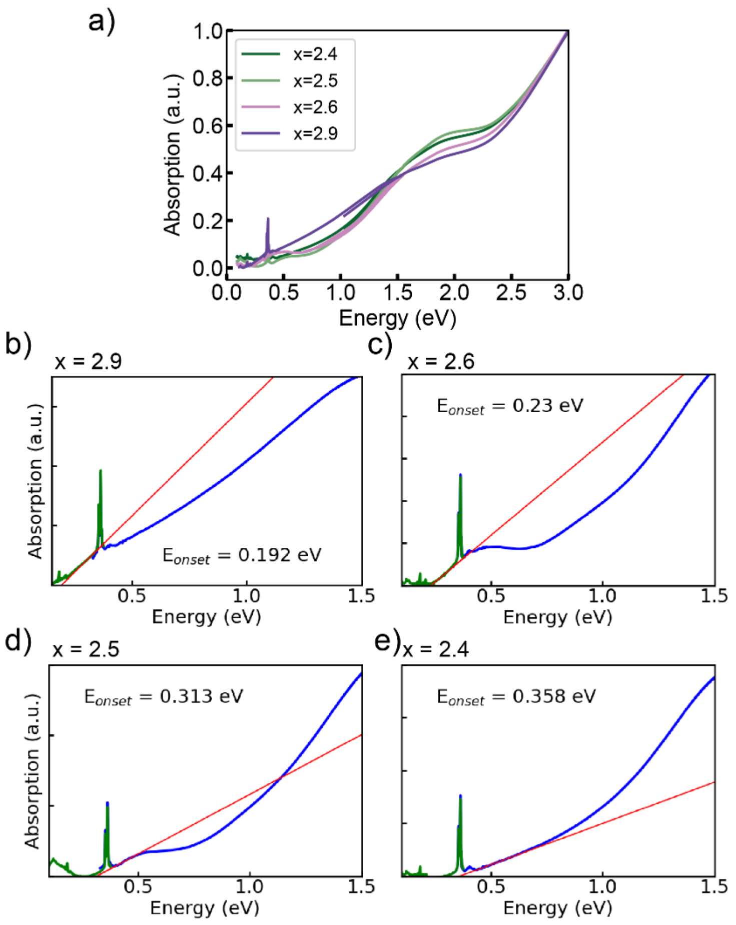

Figure S25. a) Absorption spectra over a large energy range shows an increasingly distinct shoulder around 1.8 eV for increasing Cu deficiency (smaller x in Cu_xSbSe_4 nanocrystals), suggesting a gradual change of the electronic structure possibly due to Cu vacancies and band gap widening. b)-e) FTIR spectra and absorption onset fits for solid solution Cu_xSbSe_4 nanocrystals with various x in (blue – NIR source, green – MIR source, red – linear fit).

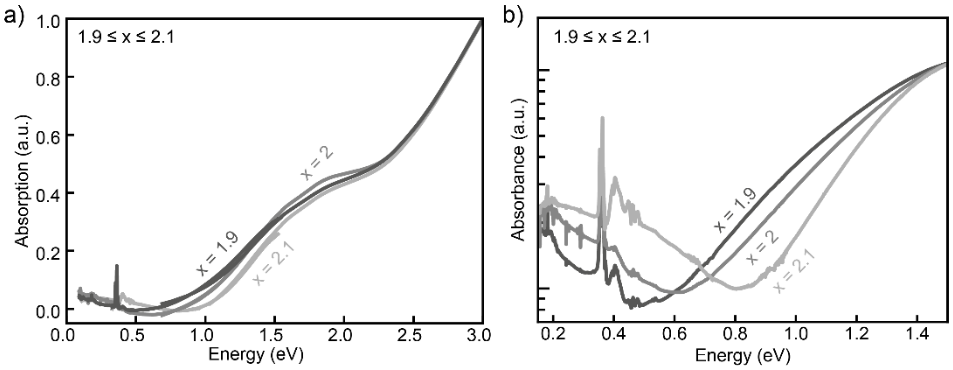

Figure S26. Trend of absorption onset of Cu_xSbSe_4 for highly Cu-deficient ($x \leq 2.1$) materials a) over a large energy range and b) showing infrared absorption in logarithmic scale. The absorption onset is between 0.5 eV and 0.9 eV and is non-zero at low energies, suggesting a high density of defect states within the band gap due to the presence of polytypism with Sb^{3+}-containing Cu-Sb-Se phase.
